# Anomaly Detection in EEG Signals: A Case Study on Similarity Measure

**DOI:** 10.1155/2020/6925107

**Published:** 2020-01-10

**Authors:** Guangyuan Chen, Guoliang Lu, Zhaohong Xie, Wei Shang

**Affiliations:** ^1^Key Laboratory of High-Efficiency and Clean Mechanical Manufacture of MOE, National Demonstration Center for Experimental Mechanical Engineering Education, School of Mechanical Engineering, Shandong University, Jinan 250061, China; ^2^Institute of Neurology, Shandong University, Jinan, China; ^3^Department of Neurology, Second Hospital of Shandong University, Jinan, China

## Abstract

*Motivation*. Anomaly EEG detection is a long-standing problem in analysis of EEG signals. The basic premise of this problem is consideration of the similarity between two nonstationary EEG recordings. A well-established scheme is based on sequence matching, typically including three steps: feature extraction, similarity measure, and decision-making. Current approaches mainly focus on EEG feature extraction and decision-making, and few of them involve the similarity measure/quantification. Generally, to design an appropriate similarity metric, that is compatible with the considered problem/data, is also an important issue in the design of such detection systems. It is however impossible to directly apply those existing metrics to anomaly EEG detection without any consideration of domain specificity. *Methodology*. The main objective of this work is to investigate the impacts of different similarity metrics on anomaly EEG detection. A few metrics that are potentially available for the EEG analysis have been collected from other areas by a careful review of related works. The so-called power spectrum is extracted as features of EEG signals, and a null hypothesis testing is employed to make the final decision. Two indicators have been used to evaluate the detection performance. One is to reflect the level of measured similarity between two compared EEG signals, and the other is to quantify the detection accuracy. *Results*. Experiments were conducted on two data sets, respectively. The results demonstrate the positive impacts of different similarity metrics on anomaly EEG detection. The Hellinger distance (HD) and Bhattacharyya distance (BD) metrics show excellent performances: an accuracy of 0.9167 for our data set and an accuracy of 0.9667 for the Bern-Barcelona EEG data set. Both of HD and BD metrics are constructed based on the Bhattacharyya coefficient, implying the priority of the Bhattacharyya coefficient when dealing with the highly noisy EEG signals. In future work, we will exploit an integrated metric that combines HD and BD for the similarity measure of EEG signals.

## 1. Introduction

In recent years, we have witnessed significant improvements of using electroencephalogram (EEG) measurement for data acquisition in a wide range of clinical applications. It has also led to the development of data mining methods that discover potential patterns in the data, aiming at characterization of dynamic EEG behaviours. Representative examples include early detection of epileptic seizure [1–3], sleep process monitoring [4–7], and many other neurological disordering related health assessment and surgery problems [8–10].

Time series is an important class of EEG data. One of its mining tasks is to detect potential anomaly event(s)/pattern(s) at an early stage in a long-term EEG monitoring process, which is highly required by change detection [11–13], seizure prediction [14, 15], etc. Hence, the notion of “*anomaly EEG detection*” is defined in the following sections.

**Figure 1:**
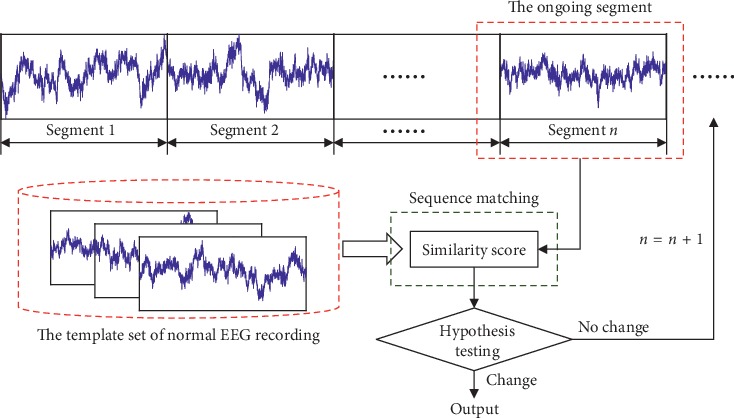
The basic premise of anomaly EEG detection.

The basic premise of anomaly EEG detection is consideration of the similarity between two nonstationary EEG recordings. A well-established scheme is based on sequence matching.  Figure 1 illustrates the computation process of this scheme. The continuously monitored EEG signal is first divided into nonoverlapping (or overlapping) segments; then, the ongoing segment under inspection is compared with those ones that are usual under normal states. It is worth noting that these normal EEG segments can be collected with a prior collection phase or directly taken from the past within the signal itself. The resulting comparison results, i.e., the similarity scores, allow for a change detection by testing a null hypothesis, *H*_0_ : *θ*=*θ*_0_ against *H*_*A*_ : *θ* ≠ *θ*_0_ on the parameters *θ* of an assumed distribution. The Gaussian distribution is the most typical assumption, and some other quantifiers, e.g., a direct threshold, can be also applicable to achieve this end. To summarize, three techniques are crucial to the success of anomaly detection, described as follows:

- *Feature Extraction*. To extract explanatory parameters from the raw EEG data in order to reduce data redundancy
- *Similarity Measure*. To employ a specific metric to measure/quantify the similarity between two data recordings, i.e., individual EEG segments
- *Decision-Making*. To make a decision by testing a null hypothesis based on the resulting similarity scores

Along this line of research, many efforts have been made to enhance the feature extraction as seen in [16–18], and some of them also involve the decision-making [4, 19, 20]. Nonetheless, we should be aware that it is also an important aspect to design an appropriate similarity metric, that is compatible with the considered data, when designing such an anomaly detection system [21]. Here, one can note that although the design of similarity metric has been an important problem in the context of statistics and data mining [22–24], the metric used for EEG signal processing still needs to be clarified due to the domain specificity. However, to the best of our knowledge, few of existing studies associated with the EEG signal processing takes into account this issue in the design of anomaly EEG detection systems.

The main objective of this work is to investigate the impacts of different similarity metrics on anomaly EEG detection based on a sequential matching scheme, which uses similarity measure coupled with a null hypothesis testing. Thus, we collect a variety of most popular and state-of-the-art metrics from other areas that would be potentially available for our problem and modify/extend them if necessary to incorporate with the anomaly EEG detection. Impacts of different metrics on anomaly detection results are evaluated based on two data sets. The experimental results reveal the different impacts of investigated metrics. Especially, the HD and BD are demonstrated outperforming performances than other competitors including PCCD, SKLD, KD, BD, and the typically used ED. This study therefore provides a preliminary basis for the EEG signal processing.

The organization of the rest of this paper is given as follows.  Section 2 formulates the considered problem.  Section 3 introduces several typical metrics that are potentially available for EEG signal analysis.  Section 4 describes the testing data and the experimental implementation.  Section 5 shows the results with some discussion.  Section 6 finally concludes this paper and shows the future work.

## 2. Problem Formulation

In this section, we first assume that the collected EEG recordings have been already represented by employed features (the feature extraction will be given in the following Section 4.2.1). We then review the method of anomaly EEG detection in the following [25].

The anomaly detection is concerned with recognising new inputs that differ in some way from those that are usual under normal states [26]. Based on this, for a given query EEG recording *x*, it is a common practice to compare it with a set of normal templates {*y*_*j*_},  *j*=1,…, *M*, where *y*_*j*_ is a EEG recording template and *M* is the total number. This size of the templates is a trade-off between sensitiveness to EEG status change and robustness to noise. If the size of the template is larger, it will be more robust to noise but less sensitive to change because the change often occurs instantaneously, and vice versa. In this paper, the size of the templates was set as 20 seconds empirically according to our clinical experience. The (anti-)similarity can be then quantified as the maximum similarity between the query recording and the templates using a similarity metric *s*. We denote it as $S\left(x\right)⟵\max_{j}s\left\{\hat{P},{\hat{Q}}_{j}\right\}$, where the $\hat{P}$ and ${\hat{Q}}_{j}$ are the features extracted from *x* and *y*_*j*_. The *x* is inspected as an anomaly event if the resulting similarity score *S*(*x*) exceeds a predefined threshold *λ*, i.e., *S*(*x*) < *λ*; otherwise, it is inspected as normal. Here, it is worth to mention that the detection can achieve a scalable and flexible detection result with using a different value of *λ*. However, since the focus of this paper is on the investigation of similarity metric, we do not make additional discussion on this issue. The interested reader can refer to [27, 28] for more discussions on this issue.

The similarity metric *s* is essential to report an accurate and reliable detection result, and its construction normally relies on a specific distance metric. A greater value of distance indicates a smaller level of similarity. More importantly, for the two given EEG recordings $\hat{P}$ and ${\hat{Q}}_{j}$, the employed distance metric needs to satisfy several fundamental properties:

- *Nonnegativity*, i.e., $s\left\{\hat{P},{\hat{Q}}_{j}\right\}\geq 0$
- *Identity*, i.e., $s\left\{\hat{P},{\hat{Q}}_{j}\right\}=0$ if and only if $\hat{P}={\hat{Q}}_{j}$
- *Symmetry*, i.e., $s\left\{\hat{P},{\hat{Q}}_{j}\right\}=s\left\{{\hat{Q}}_{j},\hat{P}\right\}$
- *Triangle inequality*, i.e., $s\left\{\hat{P},{\hat{Q}}_{j}\right\}\leq s\left\{\hat{P},\hat{R}\right\}+s\left\{\hat{R},{\hat{Q}}_{j}\right\}$, where $\hat{R}$ is a third EEG recording that is not equivalent to both $\hat{P}$ and ${\hat{Q}}_{j}$

Here, one can note that, the distance metric for similarity quantification is not necessary to meet all of these properties especially the triangle inequality, under which such kinds of distance are called as non-metric distances [29].

Based on the above definition, the similarity metric can also be confirmed as *S* ∈ [0,1] with value of 1 if two compared EEG recordings are identical and 0 if nonidentical at all. In the following, we identify some typical metrics with potentials to solving our problem by careful reviewing of the relevant literature. In particular, during the identification, two following issues were considered:

- The metric should satisfy three properties of *scalability*, *sensitivity*, and *coverage*, according to [30]
- Among various metrics, we only pay attention to the ones which only calculate the similarity between two sequences with equal lengths

## 3. Common Metrics

This section introduces a variety of metrics from other areas that would be potentially available for our problem and modify/extend them if necessary to incorporate with the considered anomaly EEG detection problem.

Let us assume that we have two sequences, *P*={*p*(*k*)},  *k*=1,2,…, *K*, and *Q*={*q*(*k*)},  *k*=1,2,…, *K*, where *p*(*k*) and *q*(*k*) are the observed values of *P* and *Q* at time *k*, respectively. A variety of typical metrics, that are potentially available for EEG analysing, are introduced to measure the similarity between *P* and *Q*.

### 3.1. Euclidean Distance (ED)

ED is the most common metric that refers to the real distance between two points in space [31]. The ED between *P* and *Q* can be calculated by

(1) $$\begin{array}{c} d^{\left(\text{ED}\right)}=\sqrt{\overset{n}{\underset{k=1}{\sum }}{\left(p\left(k\right)-q\left(k\right)\right)}^{2}}. \end{array}$$

Taking into account the characteristics of similarity metric described in Section 2, we use the reciprocal of *d*^(ED)^ to represent the similarity as

(2) $$\begin{array}{c} s^{\left(\text{ED}\right)}=\frac{1}{d^{\left(\text{ED}\right)}}. \end{array}$$

### 3.2. Pearson Correlation Coefficient Distance (PCCD)

PCCD, proposed by Pearson, is a statistic used to reflect the degree of linear correlation between two series, with values between −1 and 1. A larger value of this metric implies a stronger correlation of the two compared series [32]. The PCCD between *P* and *Q* can be calculated by

(3) $$\begin{array}{c} d^{\left(\text{PCCD}\right)}=\frac{\sum_{k=1}^{K}\left(p\left(k\right)-\bar{p}\right)\left(q\left(k\right)-\bar{q}\right)}{\sqrt{\sum_{k=1}^{K}{\left(p\left(k\right)-\bar{p}\right)}^{2}}\sqrt{\sum_{k=1}^{K}{\left(q\left(k\right)-\bar{q}\right)}^{2}}}. \end{array}$$

So, the similarity defined by PCCD is then calculated by

(4) $$\begin{array}{c} s^{\left(\text{PCCD}\right)}=\left|d^{\left(\text{PCCD}\right)}\right|. \end{array}$$

### 3.3. Symmetric Kullback–Leibler Divergence (SKLD)

SKLD can be used to measure the difference between two probability distributions, widely used in information retrieval and data science [33, 34]. The SKLD between *P* and *Q* can be calculated by

(5) $$\begin{array}{c} D\left(\left.P\right\|Q\right)=\overset{K}{\underset{k=1}{\sum }}\left(p\left(k\right)\log\left(\frac{p\left(k\right)}{q\left(k\right)}\right)\right),\\ D\left(\left.Q\right\|P\right)=\overset{K}{\underset{k=1}{\sum }}\left(q\left(k\right)\log\left(\frac{q\left(k\right)}{p\left(k\right)}\right)\right), \end{array}$$

but it is not a distance metric because of its asymmetry. In order to solve the problem, symmetric Kullback–Leibler divergence is very popular in various statistical distance metrics [35] and is calculated by

(6) $$\begin{array}{c} d^{\left(\text{SKLD}\right)}=\frac{D\left(\left.P\right\|Q\right)+D\left(\left.Q\right\|P\right)}{2}. \end{array}$$

Then, the similarity can be gotten as

(7) $$\begin{array}{c} s^{\left(\text{SKLD}\right)}=\frac{1}{d^{\left(\text{SKLD}\right)}}. \end{array}$$

### 3.4. Hellinger Distance (HD)

HD was first proposed by Hellinger in [36]. It is used in probability and statistics to measure the similarity between two probability distributions, which belongs to f-divergence [36]. The HD between *P* and *Q* can be calculated by

(8) $$\begin{array}{c} d^{\left(\text{HD}\right)}=\frac{1}{\sqrt{2}}{\left\|\sqrt{P}-\sqrt{Q}\right\|}_{2}. \end{array}$$

Thus, the similarity based on HD can be calculated as

(9) $$\begin{array}{c} s^{\left(\text{HD}\right)}=\frac{1}{d^{\left(\text{HD}\right)}}. \end{array}$$

### 3.5. Kolmogorov Distance (KD)

KD was introduced by Kolmogorov [37]. This statistical distance plays an important role in probability theory and hypothesis testing [38], and it is widely used to measure the difference between two probability distributions [39]. Therefore, the KD between *P* and *Q* can be calculated by

(10) $$\begin{array}{c} d^{\left(\text{KD}\right)}={\left\|P-Q\right\|}_{\infty }. \end{array}$$

Thus, the similarity based on KD can be calculated as

(11) $$\begin{array}{c} s^{\left(\text{KD}\right)}=\frac{1}{d^{\left(\text{KD}\right)}}. \end{array}$$

### 3.6. Bhattacharyya Distance (BD)

In the statistics, BD which was proposed by Bhattacharyya in [40], also known as the Hellinger distance, measures the similarity of two discrete or continuous probability distributions. It is closely related to the Bhattacharyya coefficient, which measures the overlap between two statistical samples or populations [23]. The Bhattacharyya coefficient can be used to determine the separability of the class classification used in the measurement of two samples that are considered relatively close. The BD between *P* and *Q* is defined as

(12) $$\begin{array}{c} s^{\left(\text{BD}\right)}=-\ln\left(BC\left(P,Q\right)\right), \end{array}$$

where *BC*(*X*, *Y*) is the Bhattacharyya coefficient.

(13) $$\begin{array}{c} BC\left(P,Q\right)=\overset{K}{\underset{k=1}{\sum }}\left(\sqrt{p\left(k\right)q\left(k\right)}\right). \end{array}$$

In the above schemes of distance metric, the similarity by some of them does not satisfy the condition *s* ∈ [0,1], as summarized in Table 1. To cope with this problem, the similarity needs to be normalized for some of them, and the normalization will be given in Section 4.2.

## 4. Materials and Methods

This section introduces the testing data and the implementation of our experiments.

### 4.1. Testing Data

**Figure 2:**
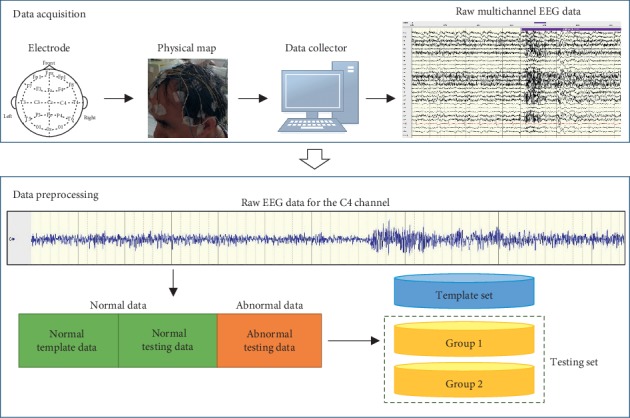
Collection of testing data based on our setup.

**Figure 3:**
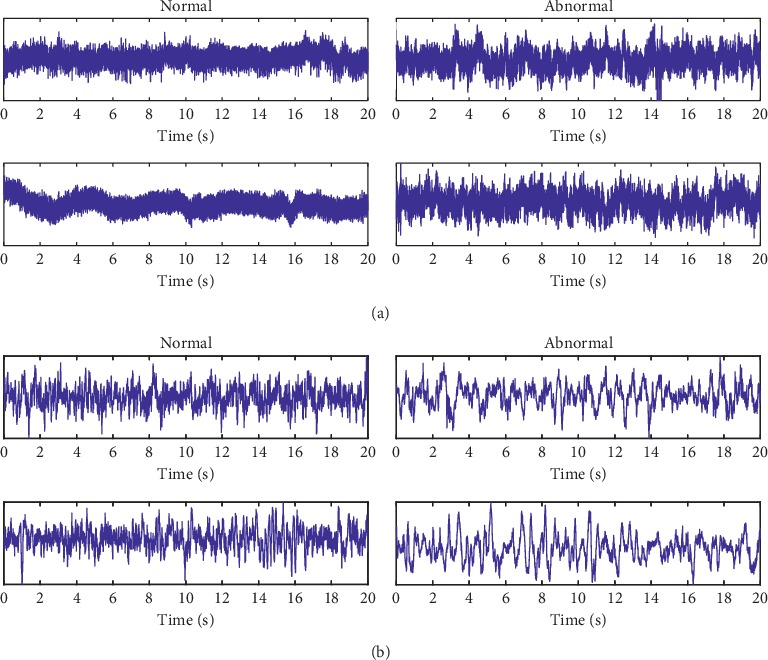
Examples of testing data: (a) collected data with our setup, and (b) data taken from Bern-Barcelona EEG database. From left to right: examples of normal data and examples of abnormal data.

The testing data in this section are from two data sets:

- The first data set is established based on our system setup. The process of data collection is depicted in Figure 2. Electrodes are placed in accordance with the International 10–20 Electrode Placement Method to collect EEG signals. The original multichannel EEG signals are obtained using the data collector. The sampling rate of data collection used here is 512 Hz. The channel C4 was chosen for our testing. Three neurological experts are invited to check the original data and label the ground-truth according to their domain experiences, i.e., which part is normal and which part is abnormal. Here, it must be pointed out that the normal status represents that the EEG signal is in a stable status, and the abnormal status includes an unstable status of the EEG signal that might be caused by seizures or other abnormal physical activities. The data are divided into several samples using a 10,000 points nonoverlapping window. Examples of tested data samples are shown in Figure 3(a).
- The second data set is taken from the public Bern-Barcelona EEG data set. They randomly select 3,750 pairs of simultaneously recorded signals from the pool of all signals measured at focal and nonfocal EEG channels, respectively, and divide the recordings into time windows of 20 seconds. The original data are recorded with a sampling rate of 1,024 Hz. Then, these EEG signals were downsampled to 512 Hz prior to further analysis so that each piece of EEG data contains 10,240 samples in length [41]. Examples of data in this data set are shown in Figure 3(b).

Additionally, for each data set, we first select 30 pieces of most table normal data segments to form a template set, and the stability and normality here are judged according to domain experts, and the residuals are as the test data. Moreover, the test data are further equally divided into two groups: one for optimizing threshold and one for final testing. Both groups contain 30 pieces of data segments, of which 15 pieces are normal data segments, and the other pieces are abnormal. The detection performance was evaluated with cross-validation of these two groups. We repeat the whole process of the evaluation twenty times, such that the final results can be obtained and analysed.

### 4.2. Experimental Implementation

Consistent with the mechanism of anomaly EEG detection introduced previously in this paper, we perform three steps, i.e., feature extraction, similarity measure, and decision-making, to carry out our experiment. Let us first denote each *i*th piece of template data as *y*_*i*_(*n*),  *n*=1,2,…, *N*, and denote each *i*th piece of testing data as *x*_*i*_(*n*),  *n*=1,2,…, *N*. Main methodologies used in the experiments are then introduced in the following.

#### 4.2.1. Feature Extraction

We extract the so-called power spectrum [21] from the raw EEG data as the feature. Let us assume that the observed value of a piece of the EEG signal at the *n*th point has been denoted as *x*(*n*), *n*=1,2,…, *N*. The EEG signal was observed in discrete situation, where the transform is discrete in both time and frequency domains [42]. We may review the discrete Fourier transform (DFT) calculation, which is formulated as

(14) $$\begin{array}{c} X\left(k\right)=\overset{N-1}{\underset{n=0}{\sum }}x\left(n\right)e^{-j\left(2\pi /N\right)kn},\;k=1,2,\ldots ,N, \end{array}$$

where *X*(*k*) is the output of the transform and *k* indicates the frequency index.

Recall that the main frequency components of EEG are *δ*-wave (<4 Hz), *θ*-wave (4–8 Hz), *α*-wave (8–14 Hz), *β*-wave (14–30 Hz), and *γ*-wave (>30 Hz) [43]. That is, if a neurological disorder happens, the amplitudes of these frequencies change accordingly. Thus, they are called characteristic frequencies; i.e., different disorders have different characteristic frequencies. Actually, many successful attempts have been reported using these frequencies to diagnose the neurological disorders [44, 45]. We hence use a subband of [0.1, 70] Hz covering these frequencies empirically for EEG inspection.

**Figure 4:**
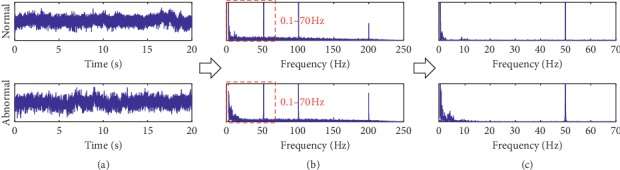
An example of feature extraction: (a) original signal; (b) DFT signal; (c) power spectrum.

After a subband passing filtering (the resulting EEG data are denoted as *x*'(*n*) after filtering), the power spectrum $\hat{P}\left(k\right)$ can be estimated using the Welch method, a typical power spectrum estimation method, by

(15) $$\begin{array}{c} \hat{P}\left(k\right)=\frac{1}{MUL}\overset{L}{\underset{i=1}{\sum }}{\left|\overset{M-1}{\underset{n=0}{\sum }}x_{n^{\prime }}^{i}\left(n\right)d_{2}\left(n\right)e^{-j\left(2\pi /N\right)kn}\right|}^{2},\;k=1,2,\ldots ,K, \end{array}$$

where *U*=(1/*M*)∑_*n*=0_^*M*−1^*d*_2_^2^(*n*) and *d*_2_(*n*) is the window function. The resulting power spectrum $\hat{P}\left(k\right)$ allows for the quantitative inspection of EEG data. An example is shown in Figure 4. It can be found that the anomaly EEG signals have the disordering amplitude variations and are polluted with a high ratio of noise. As a result, it would be very difficult to judge whether the EEG signal is abnormal through time-domain analysis. In contrast, the difference between normal and abnormal EEG signals in the frequency domain is more clear, thus allowing for quantitative inspection, i.e., similarity measure, for EEG data inspection.

Based on the above calculation of power spectrum, the testing data *x*_*i*_(*n*) and the compared template *y*_*j*_(*n*) can be represented as their corresponding power spectrums ${\hat{P}}_{i}$ and ${\hat{Q}}_{j}$, respectively.

#### 4.2.2. Similarity Measure

$s\left\{{\hat{P}}_{i},{\hat{Q}}_{j}\right\}$ is the similarity between ${\hat{P}}_{i}$ and ${\hat{Q}}_{j}$, which is calculated through the metrics described in Section 3. The similarity *S*(*x*_*i*_) of *x*_*i*_ to a normal status is thought of as the minimum *s* among all templates, i.e., $S\left(x_{i}\right)⟵\min_{j}s\left\{{\hat{P}}_{i},{\hat{Q}}_{j}\right\}$.

Furthermore, in order to satisfy the requirement described in Section 2, {*S*(*x*_*i*_)} should be normalized as [0,1] by

(16) $$\begin{array}{c} S^{\prime }\left(x_{i}\right)=\frac{S\left(x_{i}\right)-\min\left\{S\left(x_{i}\right)\right\}}{\max\left\{S\left(x_{i}\right)\right\}-\min\left\{S\left(x_{i}\right)\right\}},\;i=1,2,\ldots ,60. \end{array}$$

We still use {*S*(*x*_*i*_)} to represent the similarity for simplicity in the following.

#### 4.2.3. Decision-Making

In order to inspect whether *x*_*i*_(*n*) is normal or not, a threshold *λ* should be predefined. The decision is subsequently made by testing the following hypothesis:

(17) $$\begin{array}{c} H_{0}:S\left(x_{i}\right)>\lambda ,\\ H_{A}:S\left(x_{i}\right)\leq \lambda . \end{array}$$

If the similarity between of testing data *x*_*i*_(*n*) is greater than the threshold *λ*, the data are inspected as a normal data; otherwise, it is considered as abnormal. We first carry out a prior estimation to confirm the optimal value of *λ* with a number of EEG testing data and then use it to detect all other testing EEG signals in the experiment. The results shown in the following section are obtained by the optimal value of *λ*.

## 5. Results and Discussion

### 5.1. Experiment I: Investigation on Data Set I

As described in Section 4.1, the evaluation was repeated 20 times to obtain the final result. In the following, detailed results for one of evaluations are provided.

**Figure 5:**
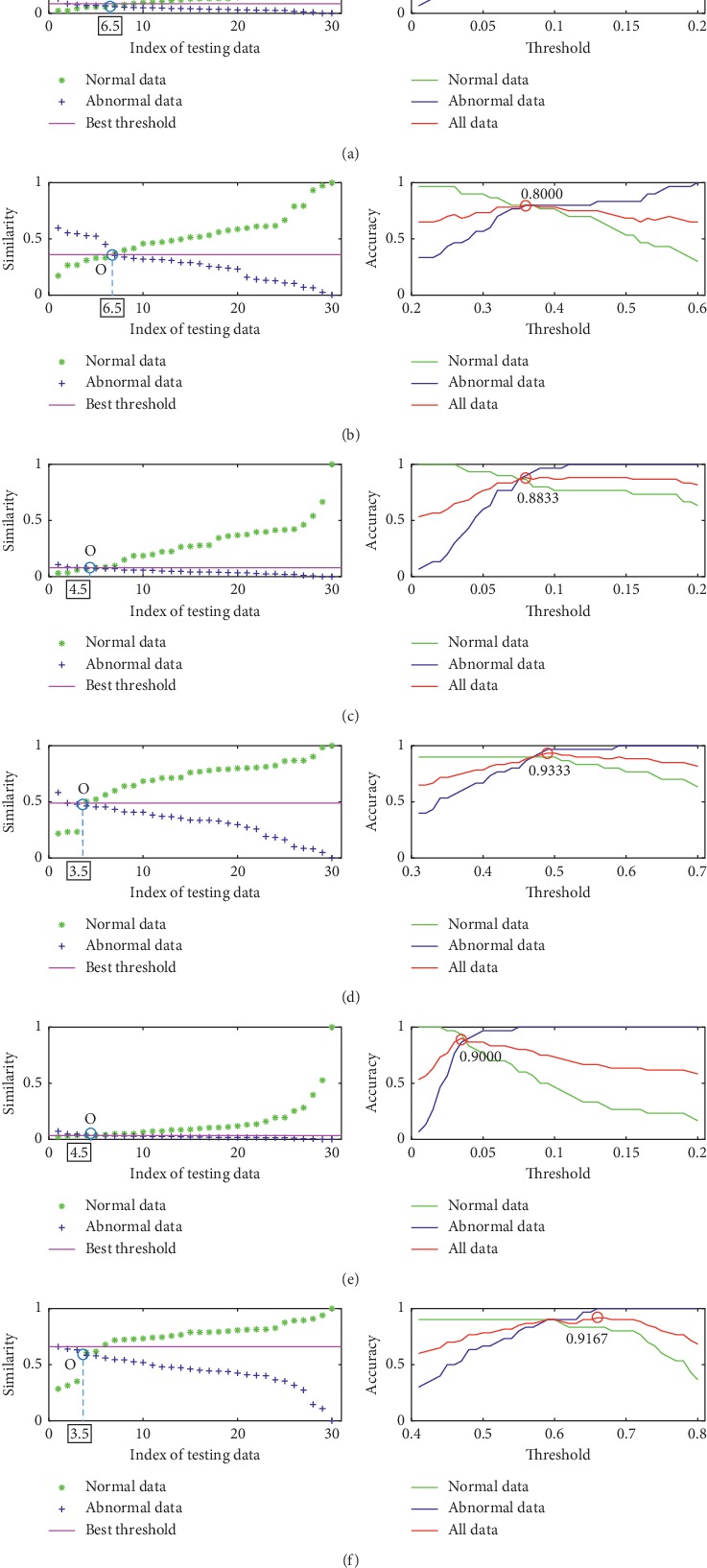
Results of six metrics using the training data of our database. From left to right: the similarity between each piece of data in the training data set and the template set and the accuracy of the metric for the normal training data, abnormal training data, and all training data. Detection result by using (a) ED, (b) PCCD, (c) SKLD, (d) HD, (e) KD, and (f) BD.

Figure 5 provides the detection results for all investigated metrics using the data of our database. In the left of each subfigure, we show the computed similarities of each training data including normal training data and abnormal training data. The similarities are gathered and then arranged in ascending order (normal testing data) or descending order (abnormal testing data). As such, two curves corresponding to normal testing data and abnormal testing data can be obtained, and they intersect at point *O*. The abscissa of point *O* (AOPO) can provide an overall evaluation for normal and abnormal testing data. A smaller AOPO means a greater difference between the normal recordings and the abnormal recordings, indicating that the similarity indicator is better; otherwise, the similarities between the two classes of recordings are not much low, meaning that the similarity indicator is not good enough. From these results, it can be clearly seen that HD and BD achieve the best result and the KD and SKLD have achieved *not-so-good* results, while the ED and PCCD have the worst results.

**Figure 6:**
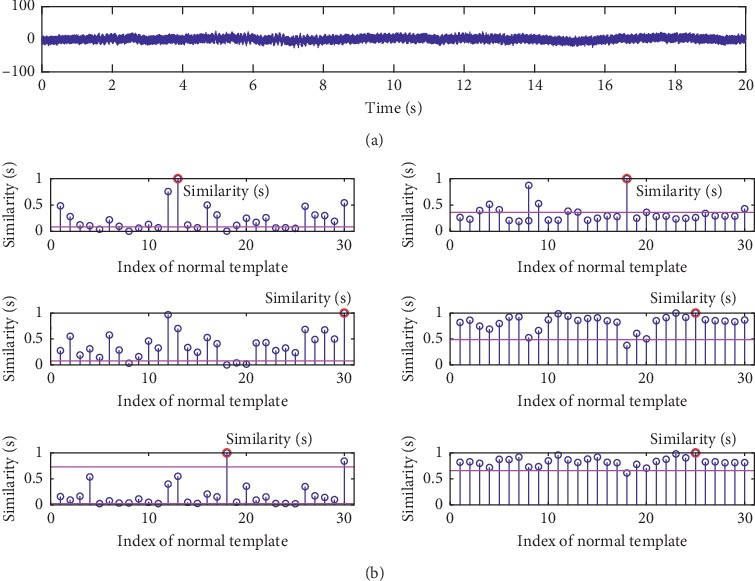
An example of similarity scores for a normal testing data in our database metrics. The similarities S between the testing data and the template set are labelled by red circles. From top to bottom: (a) the original data; (b) the similarities obtained by ED, PCCD, SKLD, HD, KD, and BD.

**Figure 7:**
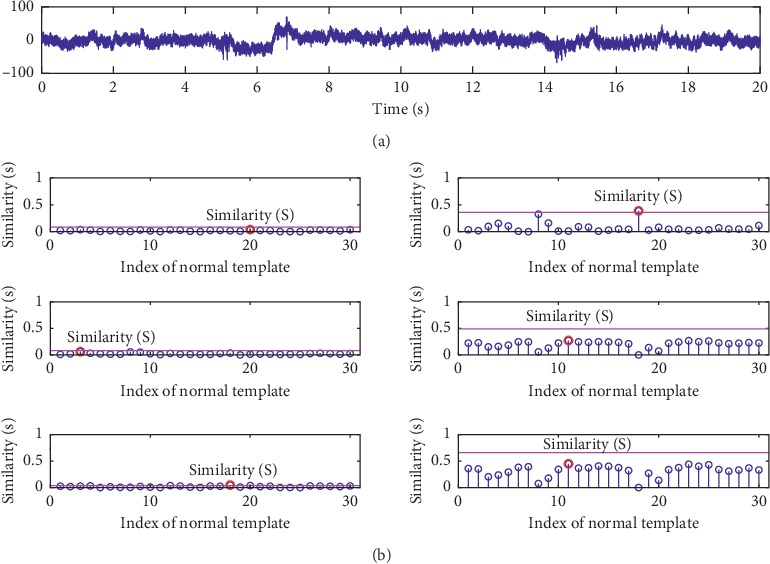
An example of the similarity scores for an abnormal testing data in our database. The similarities S between the testing data and the template set are labelled by red circles. From top to bottom: (a) the original data; (b) the similarities obtained by ED, PCCD, SKLD, HD, KD, and BD.

The other indicator of *accuracy* is also used to quantify the detection performance, which is defined as

(18) $$\begin{array}{c} accuracy=\frac{TP}{TP+FN}, \end{array}$$

where *TP* is true positive indicating the number of data that are inspected correctly and *FN* is false negative indicating the number of data that are inspected incorrectly. The right of each subfigure in Figure 5 shows the results of all metrics in term of *accuracy*. The hypothesis testing described in Section 4.2.3 is used to classify the group 1 of testing data using all investigated metrics with different threshold *λ* values. Therefore, the higher the *accuracy*, the better the metric. And it can be seen that, for each metric, as *λ* increases, *accuracy* increases first and then decreases. The values of *λ* corresponding to the highest *accuracy* are used to calculate the *accuracy* of the group 2 data set. Two examples are given in Figures 6 and 7, in which we show the similarity scores of all investigated metrics (using their optimal *λ*) for a normal testing recording and an abnormal testing recording. It can be found that PCCD and KD output wrong results for the abnormal testing data, while the others output the right results. It can be seen that the HD achieves the best performance outperforming other metrics.

We summarize the results of investigated metrics by combining their results in two terms of AOPO and *accuracy* in Table 2. It can be seen that (1) HD and BD are the best metrics in terms of AOPO and (2) HD works best in terms of *accuracy*.

The above experimental process was implemented 20 times. In order to analyse all the experimental results, we calculated the average of the AOPO and *accuracy* values obtained from all experiments based on a global mean measure and show the results in Table 3. It is noticed that the metrics of HD achieve the best performance in terms of AOPO, i.e., 3.65; in terms of *accuracy*, the HD outperforms others. Based on these results, the investigated metrics can be ranked as HD > BD > KD > SKLD > ED = PCCD.

### 5.2. Experiment II: Investigation on Bern-Barcelona Data Set

**Figure 8:**
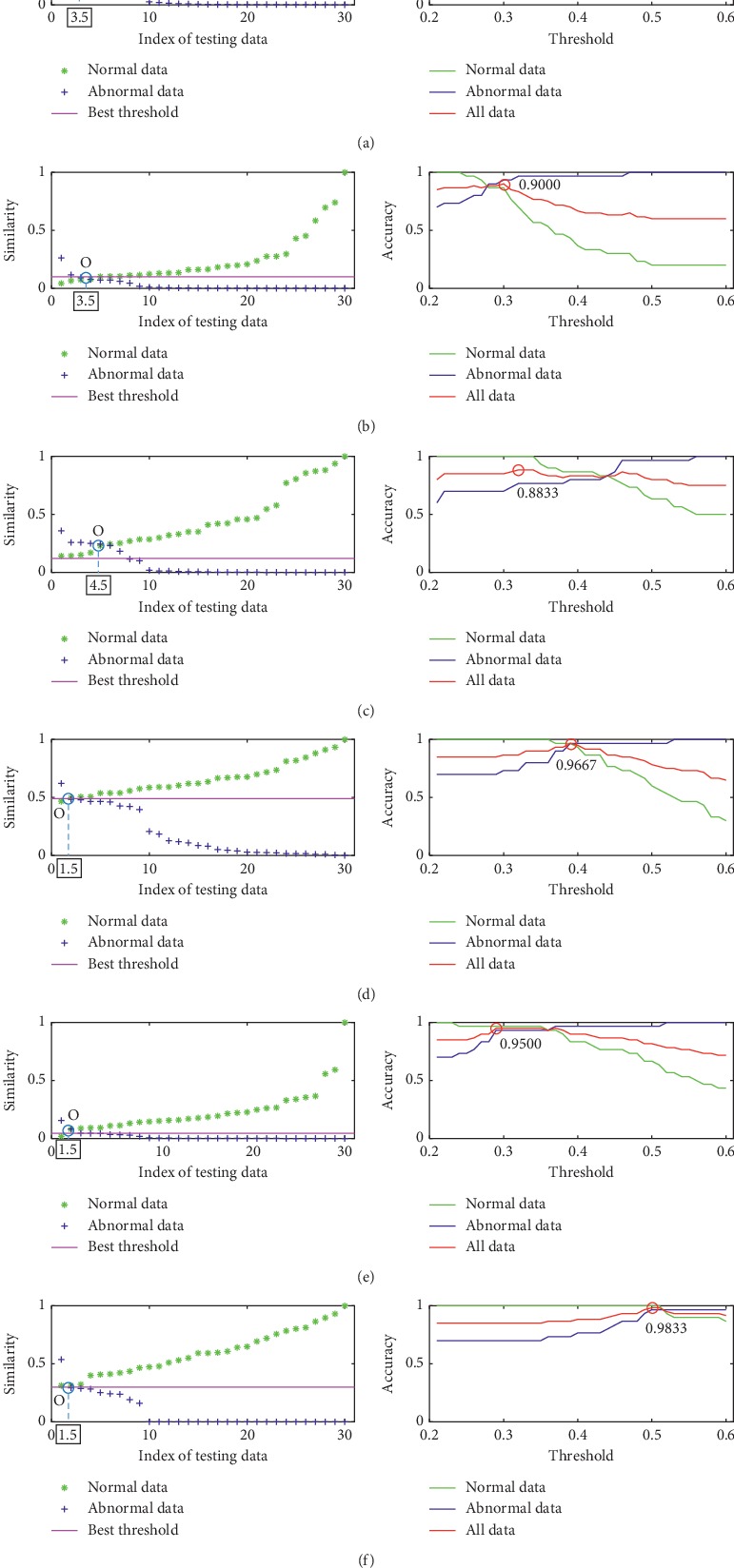
The detail results of six metrics using the training data of the Bern-Barcelona EEG database. From left to right: the similarity between each piece of data in the training data set and the template set; the accuracy of the metric for the normal training data, abnormal training data, and all training data. Detection result by using (a) ED, (b) PCCD, (c) SKLD, (d) HD, (e) KD, and (f) BD.

**Figure 9:**
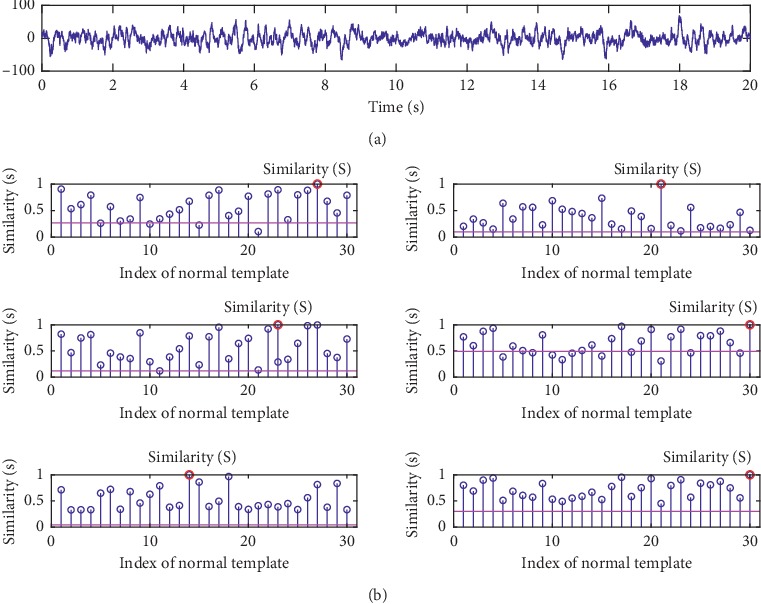
An example of similarity scores for a normal testing data of the Bern-Barcelona EEG database. The similarities S between the testing data and the template set are labelled by red circles. From top to bottom: (a) the original data; (b) the similarities obtained by ED, PCCD, SKLD, HD, KD, and BD.

**Figure 10:**
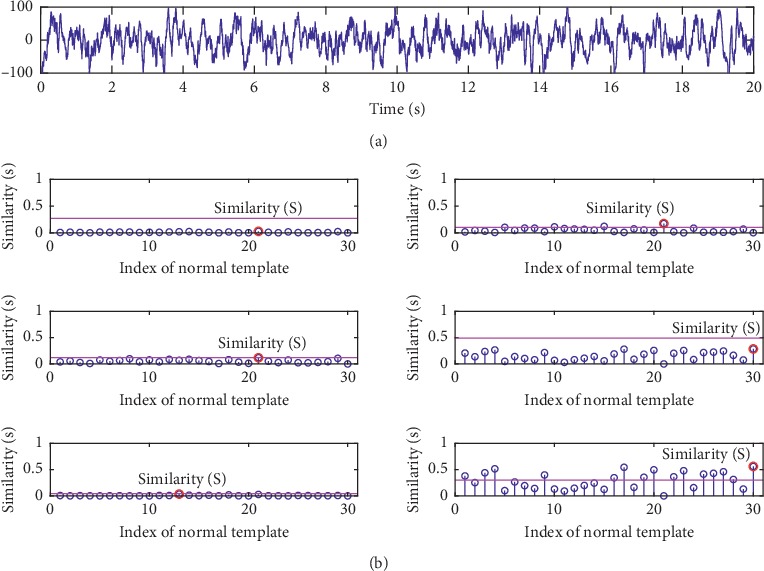
An example of similarity scores for an abnormal testing data of the Bern-Barcelona EEG database. The similarities S between the testing data and the template set are labelled by red circles. From top to bottom: (a) the original data; (b) the similarities obtained by ED, PCCD, SKLD, HD, KD, and BD.

The result of one repetitive evaluation on the Bern-Barcelona data set is also shown.  Figure 8 gives the detection results for all investigated metrics using the training data of the public Bern-Barcelona EEG database. In the left of each subfigure, we show the computed similarities of each testing data. And the similarities are also arranged in ascending order (normal testing data) or descending order (abnormal testing data). Therefore, the AOPOs in this experiment can be gotten. From these results, it can be clearly seen that HD, KD, and BD achieve the best result, the ED and PCCD have achieved *not-so-good* results, while the SKLD has the worst results. The right of each subfigure in Figure 8 shows the results of all metrics in term of *accuracy*. It is clear that, for each metric, as *λ* increases, *accuracy* increases first and then decreases too. The values of *λ* corresponding to the highest *accuracy* which is marked as *λ*_0_ are also used to calculate the *accuracy* of the group 2. Two examples are given in Figures 9 and 10, in which we show the similarity scores of all investigated metrics (using their *λ*_0_) for a normal testing recording and an abnormal testing recording. It can be found that all the metrics output the right result for the normal testing data. But for the abnormal testing data, only ED and HD output the correct result. In terms of *accuracy*, BD, HD, and HD are also better than the others.

The results of investigated metrics are also summarized in Table 4. It can be clearly seen that, in this experiment, HD, KD, and BD have achieved the best results in terms of AOPO; in terms of *accuracy*, BD works best.

The above experimental procedure was also implemented 20 times. The averages of the AOPO and *accuracy* values obtained from all experiments are shown in Table 5. Therefore, for the Bern-Barcelona EEG database, the metrics of BD achieves the best performance in terms of AOPO, i.e., 1.55; in terms of *accuracy*, the BD outperforms others. Based on these results, the investigated metrics can be ranked as BD > HD > KD > PCCD > ED > SKLD.

### 5.3. Experiment III: Investigation on Effect of Feature Extraction

In order to investigate the effect of feature extraction on detection performance, five representative features including mean, root mean square (RMS), empirical mode decomposition (EMD), discrete wavelet transform (DWT), and artifact subspace reconstruction (ASR) that are used in EEG signal analysis, are investigated in this section. Their operations are provided in Table 6. The processes of similarity measure and decision-making stated in Section 4.2 are also implemented to classify the testing data. The results of AOPO and accuracy of our database are shown in Tables 7 and 8, respectively. The results of the Bern-Barcelona EEG database are shown in Tables 9 and 10, respectively.

From the results shown in Table 7, we can see that, for our database, in terms of AOPO, the metrics of HD and BD perform better than others when using different features.  Table 8 shows the results in term of accuracy. We see that the metrics of HD and BD performs better than others when using DFT, mean, RMS, and ASR; in comparison, PCCD also shows exciting results when using the features of EMD and DWT. Tables 9 and 10 show the detection results for the Bern-Barcelona EEG database. It can be clearly seen that the metrics of HD and BD perform better than other alternatives in both terms of AOPO and accuracy.

To summarize all these results, it can be also noted that, ED, as the most commonly used indicator, performs the worst in terms of AOPO and accuracy for both testing data sets. PCCD, SKLD, and KD have achieved *not-so-good* results. Among all investigated metrics, the metrics of HD and BD are more suitable for EEG signal analysis.

### 5.4. Result Summary and Discussion

Combining the results from two tested data sets, it is clear that HD and BD achieve a better performance than the other compared metrics. Recall that both BD and HD are obtained by certain transformations of the Bhattacharyya coefficient *BC*(*P*, *Q*), i.e.,

(19) $$\begin{array}{c} s^{\left(HD\right)}=1-BC\left(P,Q\right),\\ s^{\left(BD\right)}=-\ln\left(BC\left(P,Q\right)\right). \end{array}$$

In this regard, HD and BD are thought of as an approximately equivalent measurement of two statistical samples. The difference between them is the sensitivity to noise, as discussed in [49]. However, it is very difficult to determine which of them is more appropriate for analysing the highly noisy EEG signals. As a potential solution of taking advantages of them, one can combine them using machine learning-based optimization methods, such as *inputs selection* and *inputs weighting* [50–52], to form an integrated metric to measure the considered EEG recordings. This also comprises the direction of our future work.

## 6. Conclusions

Anomaly EEG detection is a long-standing problem in analysis of EEG signals. The basic premise of this problem is consideration of the similarity between two nonstationary EEG recordings, where a well-established scheme is based on sequence matching. Typically, this scheme includes three steps: feature extraction, similarity measure, and decision-making. Current approaches mainly focus on EEG feature extraction and decision-making, and few of them involve the similarity measure/quantification. Generally, to design an appropriate similarity metric, that is compatible with the considered problem/data, is also an important issue in the design of such detection systems. It is however impossible to directly apply those existing metrics to anomaly EEG detection without any consideration of domain specificity. The main objective of this work is to investigate the impacts of different similarity metrics on anomaly EEG detection. A few metrics that is potentially available for the EEG analysis have been collected from other areas by a careful review of related works, including Euclidean distance (ED), Hellinger distance (HD), Bhattacharyya distance (BD), Kolmogorov distance (KD), Pearson correlation coefficient distance (PCCD), and Symmetric Kullback–Leibler divergence (SKLD). Experiments were conducted on two data sets to investigate them. Based on the results shown in Section 5, the following are found:

1. Experimental results demonstrate the positive impacts of different similarity metrics on anomaly EEG detection. Especially, the commonly used ED did not achieve satisfactory results when compared with other metrics. One main reason is that this metric does not consider the possibly different weight of each element in two compared EEG samples.
2. Among all investigated metrics, the HD and BD metrics, that are constructed based on the Bhattacharyya coefficient, show excellent performances. They achieved excellent performances for two inspected data sets: an AOPO value of 3.5 and an accuracy of 0.9167 for our data set and an AOPO value of 1.5 and an accuracy of 0.9667 for the Bern-Barcelona EEG data set. These findings reflect the priority of the Bhattacharyya coefficient when dealing with the highly noisy EEG signals. This study provides a preliminary basis for analysing the EEG data.

In order to take advantages of the Bhattacharyya coefficient, we will exploit an integrated metric combining HD and BD for similarity measure of EEG signals in the future work.

## Figures and Tables

**Table 1:** The range of the distance metrics.

	ED	PCCD	SKLD	HD	KD	BD
Range of distance value	[0, +*∞*]	[−1,1]	[0, +*∞*]	[0, +*∞*]	[0, +*∞*]	—
Range of similarity score	[0, +*∞*]	[0,1]	[0, +*∞*]	[0, +*∞*]	[0, +*∞*]	[0,1]

**Table 2:** The compared results of the six metrics in two ways using the testing data of our database for one experiment.

	ED	PCCD	SKLD	HD	KD	BD
AOPO	6.5	6.5	4.5	3.5	4.5	3.5
*Accuracy*	0.8167	0.7500	0.8500	0.9167	0.8667	0.9000

**Table 3:** The mean results using our database for all experiments.

	ED	PCCD	SKLD	HD	KD	BD
AOPO	6.75	6.35	4.85	3.65	4.75	3.70
*Accuracy*	0.8033	0.7583	0.8350	0.9133	0.8600	0.8933

**Table 4:** The compared results of the six metrics in two ways using the testing data of the Bern-Barcelona EEG database for one experiment.

	ED	PCCD	SKLD	HD	KD	BD
AOPO	3.5	3.5	4.5	1.5	1.5	1.5
*Accuracy*	0.9167	0.8833	0.8500	0.9500	0.9333	0.9667

**Table 5:** The mean results using the Bern-Barcelona EEG database for all experiments.

	ED	PCCD	SKLD	HD	KD	BD
AOPO	3.75	3.60	4.35	1.65	1.80	1.55
*Accuracy*	0.8833	0.8883	0.8583	0.9567	0.9250	0.9633

**Table 6:** Five compared feature extraction methods and the corresponding operations.

Name	Operation
Mean	*X*_Mean_=(1/*N*)∑_*n*=1_^*N*^*x*(*n*)
RMS	$X_{\text{RMS}}=\sqrt{\left(1/N\right)\sum_{n=1}^{N}x^{2}\left(n\right)}$
EMD	Empirical mode decomposition (EMD) is a method of signal decomposition based on the time-scale characteristics of the data itself, the detailed process of which can refer to [46].
DWT	Discrete wavelet transform (DWT) is a discrete wavelet transform method. Its detailed process can refer to [47].
ASR	Artifact subspace reconstruction (ASR) is relatively new technique, and it is based on new approach of signal reconstruction with the reference signal fragment. The detailed process of ASR can refer to [48].

**Table 7:** The AOPO results of the seven feature extraction methods using our database.

	ED	PCCD	SKLD	HD	KD	BD
DFT	6.5	6.5	4.5	3.5	4.5	3.5
Mean	14.5	13.5	10.5	7.5	10.5	8.5
RMS	10.5	8.5	7.5	4.5	6.5	5.5
EMD	9.5	7.5	7.5	5.5	7.5	4.5
DWT	9.5	6.5	8.5	4.5	6.5	6.5
ASR	7.5	5.5	4.5	3.5	5.5	3.5

**Table 8:** The accuracy results of the seven feature extraction methods using our database.

	ED	PCCD	SKLD	HD	KD	BD
DFT	0.8167	0.7500	0.8500	0.9167	0.8667	0.9000
Mean	0.5333	0.6333	0.6833	0.7833	0.7333	0.7500
RMS	0.6833	0.8833	0.7667	0.9000	0.8333	0.8833
EMD	0.7333	0.9500	0.8667	0.9000	0.8167	0.9167
DWT	0.7167	0.9833	0.7667	0.8833	0.8167	0.8500
ASR	0.8333	0.8833	0.8500	0.9000	0.8667	0.9167

**Table 9:** The AOPO results of the seven feature extraction methods using the Bern-Barcelona EEG database.

	ED	PCCD	SKLD	HD	KD	BD
DFT	3.5	3.5	4.5	1.5	1.5	1.5
Mean	13.5	9.5	8.5	7.5	8.5	5.5
RMS	7.5	6.5	6.5	4.5	5.5	2.5
EMD	10.5	6.5	4.5	3.5	5.5	3.5
DWT	9.5	5.5	7.5	4.5	6.5	3.5
ASR	7.5	3.5	4.5	1.5	6.5	2.5

**Table 10:** The accuracy results of the seven feature extraction methods using the Bern-Barcelona EEG database.

	ED	PCCD	SKLD	HD	KD	BD
DFT	0.9167	0.8833	0.8500	0.9500	0.9333	0.9667
Mean	0.5833	0.6333	0.7667	0.7833	0.7333	0.8333
RMS	0.7333	0.8833	0.8167	0.9167	0.8500	0.9333
EMD	0.6167	0.7833	0.8833	0.9167	0.8167	0.8833
DWT	0.7500	0.8333	0.8167	0.8833	0.8667	0.9000
ASR	0.7333	0.8667	0.8500	0.9333	0.7833	0.9167

## Data Availability

The data used to support the findings of this study are available from the corresponding author upon request.

## Abbreviations

AOPO: The abscissa of point O
BD: Bhattacharyya distance
DFT: Discrete Fourier transform
ED: Euclidean distance
EEG: Electroencephalogram
HD: Hellinger distance
KD: Kolmogorov distance
PCCD: Pearson correlation coefficient distance
SKLD: Symmetric Kullback–Leibler divergence Notations
*d*: The distance calculated through the metrics
*P*, *Q*: Two given probability functions
*p*(*k*): The *k*th point of series *P*
*q*(*k*): The *k*th point of series *Q*
$\hat{P}$: The power spectrum of *x*
$\hat{Q}$: The power spectrum of *y*
$\hat{R}$: An EEG recording that is not equivalent to both $\hat{P}$ and ${\hat{Q}}_{j}$
$s\left\{{\hat{P}}_{i},{\hat{Q}}_{i}\right\}$: The similarity between ${\hat{P}}_{i}$ and ${\hat{Q}}_{i}$
*S*(*x*_*i*_): The similarity between *x*_*i*_ and the template set {*y*_*i*_}
*x*(*n*): The *n*th point of a given EEG recording
*x*′(*n*): The *n*th point of resulting EEG data after filtering
*x*_*i*_: The *i*th of EEG recording of the testing data set
*X*(*k*): The *k* point of the frequency spectrum of *x*(*n*)
*y*_*j*_: The *j*th EEG recording of the template set
*λ*: The threshold used for hypothesis testing.
